# 

**Published:** 1887-03-19

**Authors:** 


### CONTENTS.

**The Public and its Physic** - - - - 411

**Words of Consolation.**—XXV. Nurses' Trials—Responsibility; Monotony - - - 412

**Nurse Farms, Hospitals, and Private Nursing Institutions.** Miss Manson's Reply - - 413

**In the Children's Ward.**—Chapter III. By Walter J. Eccott - - - - - 415

**An Umbrella for a Rainy Day.**—A National Pension Fund for Hospital Officials and Nurses - 416

**The Quilter Prizes.**—Hospital Housekeeping and Management - - - - - 417

**Notes and News.**—Miscellaneous Items.—The Baroness Burdett-Coutts and the Great Northern Central Hospital.—The East London Nursing Society.—Curious Hospital Incidents.—Vacancies.—The East London Hospital for Children.—The Royal London Ophthalmic Hospital.—The Bristol Children's Hospital.—Donations and Legacies to Medical Charities.—Children's Hospitals.—The North London Association for the Sick Poor, etc., etc. - - - 418

**Annotations.**—Teeth and their Guardians.—A Sane Woman in a Lunatic Asylum.—What is a Physician? - 421

**Hospital Patients and Medical Students.** By John MacArthur - - - - - 422

**Nursing in Workhouse Infirmaries.** By Samuel Benton, M.R.C.S. - - - - 423

**Incidents of Animal Life.**—Superstitions about Birds - - - - - - 425

**Till the Doctor Comes.**—XIII. Faintings and Fits - 426

**The Children's Corner.**—Puzzles, Answers, etc. - 427

**Amusements with Profit** - - - - 427

**In- and Out-Patients' Hospital Diary** - - 428

